# Passively Scattered Proton Therapy for Nonmelanoma Skin Cancer with Clinical Perineural Invasion

**DOI:** 10.14338/IJPT-20-00062.1

**Published:** 2021-06-25

**Authors:** Curtis M. Bryant, Roi Dagan, Adam L. Holtzman, Rui Fernandes, Anthony Bunnell, William M. Mendenhall

**Affiliations:** 1 Department of Radiation Oncology, University of Florida College of Medicine, Jacksonville, FL, USA; 2 Department of Oral and Maxillofacial Surgery, University of Florida College of Medicine, Jacksonville, FL, USA

**Keywords:** prostate cancer, radiation therapy, proton therapy, particle therapy

## Abstract

**Purpose:**

To report our experience with the delivery of passively scattered proton therapy in the management of nonmelanoma skin cancers with clinical perineural invasion.

**Materials and Methods:**

We reviewed the medical records of patients who received definitive or postoperative proton therapy for nonmelanoma skin cancer with clinical perineural invasion at our institution and updated patient follow-up when possible. All patients were treated with curative intent with or without the delivery of concurrent systemic therapy. We report disease control rates and the rates of late toxicity among this cohort.

**Results:**

Twenty-six patients treated between 2008 and 2017 were included in the analysis. Following proton therapy, the 3-year overall, cause-specific, and disease-free survival rates were 59%, 73%, and 60%, respectively. The 3-year local control, local regional control, and distant metastasis-free survival rates were 80%, 65%, and 96%, respectively. On univariate analysis, surgical resection before radiation therapy significantly improved local regional control rates at 3 years (55% versus 86%; *P* = .04). Grade 3+ late toxicities occurred in 13 patients (50%) and the most common toxicities included grade 3+ keratitis of the ipsilateral eye, which occurred in 4 patients (15%) and grade 3+ brain necrosis in 4 patients (15%).

**Conclusion:**

Proton therapy is effective in the management of nonmelanoma skin cancer with clinical perineural invasion. Although disease control and complication rates compare favorably to those previously published for photon-based radiation therapy, the risk for late toxicity is significant and patients should be appropriately counseled.

## Introduction

Nonmelanoma skin cancer (NMSC) is the most common cancer in the United States with 5.4 million cases diagnosed per year [1]. Basal cell and squamous cell carcinomas comprise more than 90% of cases and most occur within the head and neck region [1]. An estimated 1% to 5% of patients present with perineural invasion (PNI) defined by an infiltration of tumor cells along local nerves within the head and neck. These tumor cells invade the space between or under the 3 layers enclosing the peripheral nerves near the primary tumor [2–4]. Clinical PNI specifically describes cases that cause nerve deficits or can be clinically evident on radiographic examination [2].

Clinical PNI of the cranial nerves may occur with NMSC, most commonly involving the facial and trigeminal nerves [5]. Clinical PNI is both a risk factor for tumor progression after treatment and a marker for a biologically more aggressive cancer. It is associated with primary tumors that exceed 2 cm in greatest dimension, midface location, recurrent tumors, and tumors with poor differentiation. Following treatment, the presence of clinical PNI is associated with higher rates of local regional recurrence and lower overall survival following radiation therapy when compared to patients without PNI [6].

Radiation therapy is often used to treat patients with NMSC with clinical PNI. The delivery of postoperative radiation therapy to the postoperative tumor bed and the track of involved nerves leading to the skull base and/or brainstem reduces the risk for local regional recurrence. Radiation therapy can also be used definitively when tumor location and extent prohibit surgical resection. For patients with tumor involvement of the nerves within the skull base or showing intracranial invasion, radiation therapy carries the risk of delivering an excessive and potentially harmful amount of radiation to nearby neurologic structures, including the retina, optic nerves, cochlea, cerebral hemispheres, brainstem, or spinal cord. In these situations, proton therapy may help to improve the therapeutic ratio [5, 7].

When compared to x-ray radiation therapy, proton therapy reduces the amount of exit dose in the beam path beyond the treatment target. Protons deposit most of their dose at a narrow depth relative to their energy, which results in a dose distribution known as the Bragg peak. Such a reduction in excess radiation dose delivered to structures in the head and neck surrounding skin tumors with clinical PNI may help to reduce the risk for toxicities following radiation therapy, including retinopathies, optic neuropathies, hearing loss, and brain or brainstem necrosis [8]. Because of the relative biologic effectiveness (RBE) of proton therapy, it is predicted to be at least as effective in treating cancer as photon-based radiation therapy [7, 8]. Below we report our institutional outcomes using proton therapy in the management of patients with nonmelanoma skin cancers with clinical PNI and summarize the important principles of managing this rare but aggressive disease.

## Materials and Methods

We reviewed patient medical records and updated follow-up, part of an institutional review board–approved outcomes-tracking protocol allowing for prospective assessment of toxicity and disease status among patients receiving proton therapy between 2008 and 2017; some patient data were collected with informed consent and some under a waiver of informed consent. We identified patients with a diagnosis of biopsy-proven NMSC with clinical PNI of a named major cranial nerve or one of its branches. All patients had skin cancers within the head and neck region. Perineural invasion was confirmed pathologically and/or radiographically with a diagnostic magnetic resonance image of the head and neck. Patients required at least 1 year of potential follow-up for inclusion in the analysis. Staging was performed according to the *American Joint Committee on Cancer Staging Manual*, 8th edition [9]. Complications were graded prospectively by using the Common Terminology Criteria for Adverse Events, version 4 (US National Cancer Institute Bethesda, MD). Local recurrence was defined as recurrence of disease in areas included in the high-risk clinical target volume, which encompassed gross disease along the skin, bone, soft tissue, or nerves at the time of diagnosis. Regional recurrence was defined as disease recurrence within at-risk nodal stations or contiguous nerves within the head and neck that were not thought to be involved at the time of diagnosis and were not included in the high-risk clinical target volume.

Proton therapy was delivered definitively after a biopsy of gross disease or postoperatively after a surgical resection of gross disease. In general, postoperative radiation therapy was recommended at our institution following surgical resection of NMSC with clinical PNI to lower the risk for local regional recurrence. Proton therapy was delivered by using standard fractionation at 1.8 to 2 GyRBE per fraction to a preferred total dose of 70 GyRBE to gross disease. Alternatively, hyperfractionated proton therapy was delivered at 1.2 GyRBE twice daily to a total dose ranging from 72 to 74.4 GyRBE. This fractionation scheme was delivered when optic structures, including optic nerves, chiasm, or retina, were close to the planning target volumes. Previous publications have shown that hyperfractionation may lower the risk for optic neuropathy, retinopathy, and auditory dysfunction following high-dose radiation therapy when compared to standard fractionation for skull base tumors [10, 11]. Passively scattered proton therapy was delivered to all 26 patients. Elective regional nodal radiation was recommended for at-risk nodal stations and it was delivered either with proton therapy or by using a separate matched electron or photon radiation field. In all cases, passively scattered techniques were used to deliver proton radiation to the primary site of disease and any areas near vital neurologic structures. Although individual physician discretion was used to develop target volumes, in the case of gross nerve involvement, our standard has been to include the gross disease plus a 0.5- to 1-cm margin edited for anatomic boundaries as the high-risk clinical target volume (CTV HR). Any gross disease involving the skin or subcutaneous tissues was also included in this volume, using the same margins. This volume received the prescription dose of radiation therapy ranging from 70 to 74.4 GyRBE at 1.8 to 74.4 GyRBE depending on fractionation scheme. The standard-risk clinical target volume (CTV SR) included the involved nerve to the base of skull, nerve root, or ganglion depending on the deepest extent of radiographic tumor invasion. Radiographically uninvolved cranial nerve branches known to anatomically have direct communication to the involved nerve and ipsilateral regional nodes including periparotid nodes were also included in the CTV SR in appropriate cases. This volume was typically treated at 50 to 50.4 GyRBE depending on fractionation. Concurrent chemotherapy was given per physician discretion and usually consisted of concurrent weekly cisplatin delivered at 30 mg/m^2^.

JMP software was used for tabulations and statistical analysis (SAS Institute, Cary, North Carolina). The Kaplan-Meier product limit method provided estimates of local control, local regional control, and overall survival. The log-rank test statistic was then applied to the Kaplan-Meier estimates to assess whether there were any statistically significant differences in these outcomes when stratifying by surgical resection, concurrent chemotherapy, or tumor location. To assess whether chemotherapy had any impact on grade 3+ keratitis or brain necrosis rates, Fisher exact test was used as the nonparametric analog to the likelihood ratio χ^2^ test given that some cells in the contingency tables had cell counts below 5. For all statistical tests, the threshold for determining statistical significance was α = .05.

## Results

The study cohort was composed of 26 patients treated between 2008 and 2019. Table 1 summarizes patient clinical characteristics, including the subsites of initial disease location. Most patients were male (21; 81%) and most had a pathologic diagnosis of squamous cell carcinoma of the skin (23; 88%). Among 18 patients, the development of PNI was believed to be a manifestation of tumor recurrence following a previous excision of a nonmelanoma skin cancer of the head and neck. Nineteen patients (73%) had 1 cranial nerve involved with cancer at the time of treatment and 7 (27%) had multiple nerves involved. The deepest extent of invasion was to the skull base in 10 patients (38%) and intracranially in 9 patients (35%). Only 1 patient in this cohort was immunosuppressed at the time of treatment. This patient had previously received a kidney transplant and was taking immunosuppressant medications.

**Table 1.** Patient treatment characteristics.

**Characteristic**	**No. of patients (%)**
Age, median (range), y	67 (51-80)
Race	
Caucasian	26 (100)
Sex	
Male	21 (81)
Female	5 (19)
Clinical T stage	
T4	26 (100)
Nodal stage	
N0	24 (92)
N2	2 (8)
Histology	
Squamous cell carcinoma	23 (88)
Basal cell carcinoma	2 (8)
Poorly differentiated carcinoma	1 (4)
De novo or recurrent	
De novo	8 (31)
Recurrent after initial resection	18 (69)
Major named cranial nerves grossly involved	
Trigeminal	18 (69)
Facial	1 (4)
Trigeminal and facial	7 (27)
Number of major named nerve branches involved (VII, V1, V2, V3)	
1	19 (73)
2	4 (15)
3+	3 (12)
Deepest proximal extent of nerve invasion	
Distal	7 (27)
Skull base	10 (38)
Intracranial	9 (35)
Site of primary tumor	
Forehead/temple	8 (31)
Unknown	6 (23)
Cheek	2 (8)
EAC	3 (12)
Eyelid	2 (8)
Nose	5 (19)

**Abbreviation:** EAC, external auditory canal.

Treatment characteristics are described in Table 2. A gross total resection with negative margins was accomplished for 4 patients (15%). The remaining patients had either a resection with positive microscopic margins (3 patients, 12%) or, because of the tumor location and invasiveness, a biopsy at the time of diagnosis only (19 patients, 73%). All patients were treated with proton therapy to a median dose of 72 GyRBE (range, 68.4-74.4 GyRBE). Twice-daily radiation therapy using 1.2-GyRBE fractions was delivered to 21 patients (81%). Elective nodal radiation therapy was performed in 25 patients (96%). Concurrent chemotherapy was delivered in 16 patients (62%) in the form of weekly cisplatin or carboplatin. Typically, low doses of cisplatin were used at 30 to 40 mg/m^2^ per dose.

**Table 2.** Treatment characteristics.

**Characteristic**	**No. of patients (%)**
Radiation therapy treatment	
Definitive radiation therapy	19 (73)
Postoperative radiation therapy	7 (27)
Elective nodal radiation therapy	25 (96)
Delivered using proton therapy	6 (23)
Delivered with matching 3D conformal photon field	11 (43)
Delivered using en face electron field	6 (23)
Delivered using IMRT	2 (8)
Fractionation	
Twice daily, 1.2-GyRBE fractions	21 (81)
Once daily, 1.8- to 2-GyRBE fractions	5 (19)
Total radiation therapy dose, median (range), GyRBE	72.2 (68.4-74.4)
Extent of surgery before radiation therapy	
Gross total resection (R0, meaning a grossly resected tumor with negative microscopic margins)	4 (15)
Grossly resected tumor with positive microscopic margins (R1)	3 (12)
Biopsy only	19 (73)
Concurrent chemotherapy with radiation therapy	16 (62)

**Abbreviations:** 3D, three-dimensional; IMRT, intensity-modulated radiation therapy.

**Figure 1.**
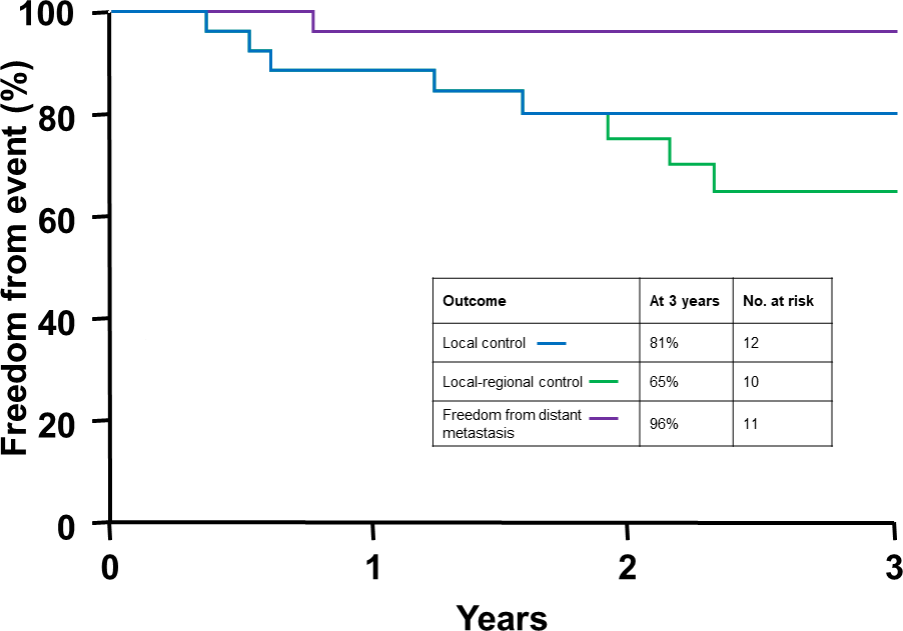
Kaplan-Meier curves depicting survival rates among the cohort at 3 years (N = 26).

**Figure 2.**
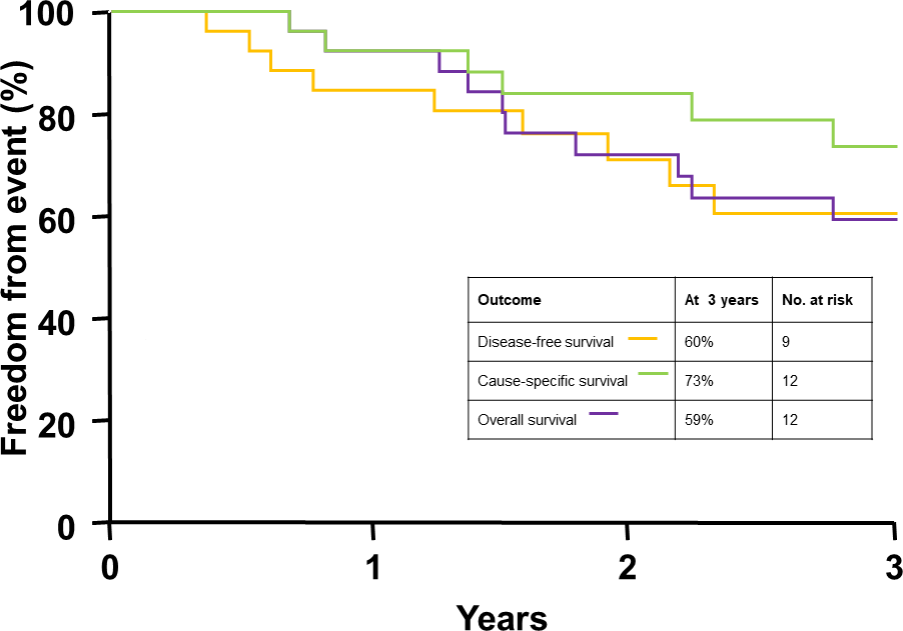
Kaplan-Meier curves depicting disease control rates among the cohort at 3 years (N = 26).

The median follow-up for the entire cohort was 2.8 years (0.7-11.5 years), and for patients who were alive at last follow-up it was 4.0 years (1.0-11.5 years). The median time to death for patients who were deceased at last follow-up was 1.6 years (range, 0.7-9.3 years). As Figure 1 shows, overall survival, cause-specific survival, and disease-free survival rates at 3 years were 59%, 73%, and 60%, respectively. Local control, local regional control, and freedom from distant metastasis rates were 80%, 65%, and 96% at 3 years, respectively, as seen in Figure 2. The initial sites of failure were as follows: local, 7 (27%); regional nerves, 3 (12%); regional soft tissues, 2 (8%); regional nodal, 1 (4%); and distant metastasis, 1 (4%). The median time to local failure, regional failure, and local regional failure was 1.2 years (range, 0.4-9.2 years), 1.7 years (range, 0.6-2.3 years), and 1.7 years (range, 0.4-9.2 years). Both patients with basal cell carcinoma are disease-free at last follow-up, whereas 46% of patients with squamous cell carcinoma experienced disease recurrence.

Table 3 provides the results of the univariate analyses regarding local control, local regional control, and cause-specific survival. Surgical resection of gross disease before radiation therapy appeared to improve 3-year local regional control, which was 55% for those who had a biopsy only versus 86% for those who had a surgical resection (*P* = .04). It also appeared to improve 3-year local control (72% versus 100%, *P* = .05). The delivery of concurrent chemotherapy with radiation therapy, the timing of the disease presentation, and the most proximal extent of clinical PNI did not significantly impact local control, local regional control, or cause-specific survival.

**Table 3.** Univariate analysis for local control, local regional control, and cause-specific survival.

**Factors**	**3-year rates, %**	***P*** **value**
Local control		
Surgical resection, yes versus no	100 vs 72	.05
Concurrent chemotherapy, yes versus no	75 vs 89	.24
Proximal extent of perineural invasion, intracranial versus skull base versus distal	89 vs 70 vs 86	.20
De novo versus recurrent disease	76 vs 82	.85
Local regional control		
Surgical resection, yes versus no	86 vs 55	.04
Concurrent chemotherapy, yes versus no	57 vs 76	.19
Proximal extent of perineural invasion, intracranial versus skull base versus distal	53 vs 70 vs 71	.46
De novo versus recurrent disease	61 vs 66	.83
Cause-specific survival		
Surgical resection, yes versus no	86 vs 69	.18
Concurrent chemotherapy, yes versus no	72 vs 76	.71
Proximal extent of perineural invasion, intracranial versus skull base versus distal	89 vs 70 vs 71	.71
De novo versus recurrent disease	58 vs 82	.66

A total of 13 patients (50%) experienced a total of 16 (62%) grade 3 complications, which are described in Table 4. The most common grade 3+ complication was keratitis, which occurred in 4 patients (15%). Each patient had keratitis that limited vision or caused blindness in the affected eye. The median time to development of keratitis was 23 months (range, 5-31 months). The rate for grade 3+ keratitis for those who had received concurrent systemic therapy versus those who did not was 19% (3 of 16) and 10% (1 of 10), *P* = .99, respectively. The median of the maximum dose of radiation delivered to the globe of the eyes damaged by radiation therapy was 75 GyRBE (range, 67-79 GyRBE). The second most common late grade 3+ toxicity was brain necrosis, which occurred at a median time of 23 months (range, 19-102 months) in 4 patients; each patient had received 70 GyRBE to areas that later developed necrosis and each patient had received concurrent weekly cisplatin during radiation therapy. The rate of grade 3+ brain necrosis for those who had received concurrent systemic therapy versus those who had not was 25% (4 of 16) versus 0% (0 of 10; *P* = .13). Patients with brain necrosis were all symptomatic and required medical intervention beyond corticosteroids.

**Table 4.** Late grade 3+ toxicity per National Cancer Institute Common Terminology Criteria for Adverse Events, version 4.

**Toxicity**	**Grade 3+, n (%)**
Keratitis	4 (15)
Hearing loss	2 (8)
Brain necrosis	4 (15)
Orbital infection	1 (4)
Osteoradionecrosis	1 (4)
Hematoma	1 (4)
Cataract	1 (4)
Soft tissue necrosis	2 (8)

## Discussion

NMSCs that demonstrate clinical PNI present a difficult challenge for head and neck oncologists. Effective treatment often requires multimodal strategies involving surgery, radiation therapy, and chemotherapy and the proximity of these tumors to critical neurologic structures, including the eyes, cranial nerves, brainstem, and cerebral hemispheres, places patients at risk for serious complications following treatment. One way to potentially mitigate the risk for serious side effects following radiation is to deliver proton therapy, which can lessen the amount of excess radiation therapy delivered to organs at risk near the skull base when compared to photon-based radiation therapy.

In this series, we present results from a cohort of patients with clinical PNI treated with proton therapy. Patients could have received definitive or postoperative radiation therapy. Concurrent chemotherapy with radiation therapy was given per physician discretion. When compared to other series evaluating the role of definitive photon-based radiation therapy for this disease, proton therapy compares favorably, as shown in Table 5 [5, 12–15]. For example, Balamucki et al [5] reviewed the outcomes of 109 patients treated with photon-based radiation therapy for nonmelanoma skin cancer with clinical PNI. Most patients had squamous cell carcinomas (81%) and patients were treated to a median dose of 70.2 Gy. At 5 years of follow-up, the overall survival, cause-specific survival, and freedom from distant metastasis rates were 54%, 64%, and 94%, respectively. A total of 39 patients (36%) developed a grade 3 late complication and the most common complications were blindness (11%) and bone exposure (11%). Although these rates are lower than those reported in our study, toxicity was scored in a retrospective manner, which may underestimate the risk for complications following radiation therapy [5].

**Table 5.** Literature review.

**Study author, year [reference]**	**No. of patients**	**Surgical excision, %**	**Therapy**	**Median dose, Gy**	**Median follow-up, y**	**Disease-free survival**	**Toxicity grade 3+, %**
Balamucki et al, 2012 [5]	109	56	Photon radiation	70.2	4	51% at 5 y	36
Lin et al, 2013 [12]	56	73	Photon radiation	60	NR	48% at 5 y	NR
Warren et al, 2016 [13]	50	100	Photon radiation	50-63	4.2	75% at 5 y	NR
Sapir et al, 2016 [14]	35	77	Photon radiation	66	1.8	64% at 2 y	NR
Chen et al, 2018 [15]	23	65	Photon radiation	65-70	2.6	29% at 2 y^a^	NR
Present study	26	29	Proton radiation	72	2.8	61% at 3 y	50

**Abbreviation:** NR, not recorded.

a Local regional control.

Chen et al [15] published a series of 23 patients with NMSC with clinical PNI who were treated with photon-based external-beam radiation therapy. Doses of 60 to 70 Gy were delivered with standard fractionation. Fifteen patients (65%) had surgical resection before radiation therapy and 16 patients received concurrent chemotherapy with radiation therapy. Local regional control and cause-specific survival were 29% and 25% at 2 years, respectively. Toxicity rates were not reported.

Warren et al [13] published a series of 50 patients with NMSC with clinical PNI. All patients were treated with surgical resection followed by postoperative photon-based radiation therapy. Patients were treated to a total dose ranging from 50 to 63 Gy delivered with standard fractionation. At 5 years, the recurrence-free survival rate was 62%, while the disease-specific and overall survival rates at 5 years were 75% and 64%, respectively. These relatively promising results suggest surgical resection should be recommended when feasible as it may impact recurrence-free survival [13]. Our series showed that surgical resection improved local control and local regional control, which supports these findings.

Lin et al [12] published a series including 56 patients with head and neck NMSC with clinical PNI. All patients received either definitive (23%) or postoperative (73%) photon-based radiation therapy. Patients were treated to a median radiation therapy dose of 60 Gy (range, 48-74 Gy). The recurrence-free survival rate at 5 years was 48% overall. Surgical resection before radiation therapy did not significantly affect recurrence rates (45% versus 59%, *P* = .46) but because of small patient numbers this study may not have had enough power to detect a clinically meaningful difference with this comparison.

Our series showed that the most common site of regional recurrence was along the cranial nerve branches in direct communication with the involved nerve. Gluck et al [16] published similar findings in a series of 11 patients treated with photon-based radiation therapy. Patients were treated to the involved skin and nerves at the time of presentation and 8 of 11 patients developed disease recurrence in previously untreated but contiguous nerves within the head and neck. This was the most common location of recurrence and is representative of the natural history of NMSC with clinical PNI. Because of this finding and results from our series, we recommend including contiguous nerves within the standard-risk clinical target volume to reduce the risk for this type of regional recurrence.

The risk for complications is high with radiation therapy to the skull base. When delivering high doses (70 GyRBE) to neurologic structures including the cerebral hemispheres, brainstem, cochlea, and optic nerve structures of the eye, complications are expected. Half of the patients in the cohort experienced a grade 3 complication. The most common were keratitis leading to decreased vision in one eye and brain necrosis. All patients who developed grade 3+ brain necrosis also received concurrent cisplatin during radiation therapy. Although several studies have indicated that the receipt of cytotoxic chemotherapy increases the risk for brain necrosis when delivering stereotactic brain radiosurgery and whole-brain radiation therapy, this is an area that requires further prospective assessment among patients being treated with fractionated radiation therapy for base of skull tumors [17, 18].

Although proton therapy delivers less excess radiation to nearby tissues than photon-based radiation therapy, the risk is still present as gross tumor is often immediately adjacent to or overlaps with critical neurovascular structures. In our experience, pencil beam scanning (PBS) is a more conformal form of proton therapy than passively scattered techniques. Iwata et al [19] have recently shown that when treating head and neck cancer, PBS compared to passively scattered proton therapy improved the conformality index, conformality number, and homogeneity index. On average, PBS also provided lower doses to the brain, eye, optic nerves, and optic chiasm. Because all of our patients were treated with passively scattered proton therapy, our experience may overestimate the risk for complications that might be expected with proton therapy delivered with PBS. The risk for complications with proton therapy will likely be lower as PBS becomes the standard method of proton therapy delivery for this malignancy.

The limitations of this study include its relatively small size, short follow-up times, and the use of multiple types of radiation therapy. Only 26 patients were included in the analysis, which limits the power to identify factors that may affect survival and disease control outcomes, as a multivariate analysis cannot be performed adequately. Additionally, the median follow-up was only 2.8 years, which limits the potential to accurately report late toxicities. Some late toxicities, such as cranial nerve neuropathies, can occur 3 or more years after radiation therapy to the skull base [20, 21]. Finally, proton therapy was delivered with passively scattered proton therapy. In addition, some patients had elective nodal radiation therapy delivered with 3-dimensional conformal photon- or electron-based radiation therapy. These less conformal techniques and the heterogeneity of the delivery of radiation therapy make it difficult to form valid conclusions about the efficacy and safety of modern-day proton therapy, as technology has improved over time.

## Conclusions

Proton therapy has a role in the management of patients with NMSC with clinical PNI of the head and neck. It effectively delivers either definitive or postoperative irradiation with or without concurrent systemic therapy. The risk for regional recurrence is high following treatment, especially for cranial nerves that are contiguous with the primary nerve involved with the tumor. We recommend delivering elective radiation therapy to contiguous nerves and nerves that innervate or supply sensation to the skin involved with the primary tumor. The risk for complications following radiation therapy is high, particularly for the ipsilateral eye, cochlea, and nearby brain structures, which often receive radiation doses in excess of their tolerance. Delivering proton therapy with PBS may lower the risk of complications in future patients. Because of the potential for long-term disease control with radiation therapy, we recommend counseling patients about the risks before delivering radiation therapy, and exceeding goals when necessary to provide a curative dose to gross disease within the skull base and intracranial structures.
